# Soluble CD40 Ligand Levels in Children with Newly Diagnosed Graves’ Disease

**DOI:** 10.4274/jcrpe.galenos.2019.2019.0108

**Published:** 2020-06-03

**Authors:** Kotb Abbass Metwalley, Hekma Saad Farghaly, Duaa Mohammed Raafat, Asmaa Mohammed Ismail, Ghada Mohamed Saied

**Affiliations:** 1 Assiut University Faculty of Medicine, Department of Pediatrics, Assiut, Egypt; 2 Aswan University Faculty of Medicine, Department of Pediatrics, Aswan, Egypt; 3 Assiut University Faculty of Medicine, Department of Clinical Pathology, Assiut, Egypt

**Keywords:** Graves’ disease, soluble CD40 ligand (sCD40L), thyroid hormone, thyroid volume

## Abstract

**Objective:**

Soluble CD40 ligand (sCD40L) is elevated in various autoimmune disorders, which may have diagnostic and therapeutic implications. The aims of the current study were to evaluate serum sCD40L concentrations in children with newly diagnosed Graves’ disease (GD) and to correlate its levels with patients’ clinical and laboratory parameters.

**Methods:**

This study included 48 children with newly diagnosed GD and 48 healthy children. Serum thyroid-stimulating hormone (TSH) (TSH, fT4 and fT3), TSH receptor antibodies (TRAbs), high sensitivity C-reactive protein (hsCRP) and sCD40L levels and thyroid volume were measured.

**Results:**

Compared to control subjects, children with GD had higher thyroid volume standard deviation scores (SDS) (p=0.001), and higher levels of hsCRP (p=0.001), TRAbs (p=0.001) and sCD40L (p=0.001). Significant correlations were found between sCD40L and age (p=0.01), thyroid volume SDS (p=0.001), hsCRP (p=0.01) and TRAbs (p=0.001). In multivariate analysis, sCD40L concentrations were correlated with TRAbs [odds ratio (OR)=3.1, 95% confidence intervals (CI): 2.2-2.7, p=0.001] and thyroid volume SDS (OR=2.1, 95% CI: 1.2-2.7, p=0.001).

**Conclusion:**

This preliminary study has evidence of high concentrations of sCD40L in children with newly diagnosed GD and a correlation between sCD40L and both TRAbs and thyroid volume, which may indicate a biologically active role for sCD40L in the pathogenesis of GD.

**What is already known on this topic?**

Graves’ disease (GD) is believed to result from a complex interaction between genetic background, environmental factors, and the immune system. Soluble CD40 ligand (sCD40L) might be involved in the evolution of many autoimmune diseases and may have diagnostic and therapeutic implications.

**What this study adds?**

To our knowledge, this is the first study to assess serum sCD40L concentrations in children with newly diagnosed GD. High concentrations of sCD40L were found in children with newly diagnosed GD compared to healthy controls and there was a correlation between sCD40L and thyroid stimulating hormone receptor antibodies and thyroid volume which may suggest a biologically active role for sCD40L in GD.

## Introduction

Graves’ disease (GD), the most common cause of spontaneous thyrotoxicosis, is believed to result from a complex interaction between genetics, environmental factors, and the immune system (1). GD is mediated by autoantibodies against the thyroid stimulating hormone (TSH) receptor (TRAbs) that bind to and activate TSH receptors, thus stimulating thyroid hormone synthesis, secretion and thyroid cell growth (2). Cluster of differentiation 40 ligand (CD40L) is a trimeric transmembrane protein of the tumor necrosis family and was originally identified on the cells of the immune system (3). It binds to CD40, which is mainly expressed on antigen-presenting cells and B cells although it is present on other types of cells such as thyroid follicular cells (4). After cellular binding, the surface-expressed CD40L is then cleaved and/or released over a period of minutes to hours generating a soluble fragment (sCD40L), which retains full biological activity. It has number of immune functions that include cell-to-cell interactions, antigen presentation and pathogen capture (5). CD40-sCD40L interaction has an emerging role in the evolution of some autoimmune diseases such as systemic lupus erythematosus, rheumatoid arthritis and mixed connective tissue disease (6). Little is known about the role of sCD40L in GD (7). This study was conducted as a preliminary evaluation to estimate the serum concentrations of sCD40L in a group of children with newly diagnosed GD and its relationship to patients’ clinical and laboratory variables.

## Methods

### Patients

This is a cross-sectional case-control study involving children and all were newly diagnosed before the start of medical treatment. They were consecutively recruited over a period of two years from 2015 to 2017 and all were attending the Pediatric Endocrinology Clinic of Children’s Hospital, Assiut University, Assiut, Egypt. The diagnosis of GD was based on the presence of clinical manifestations of hyperthyroidism, low serum levels of TSH, high serum levels of free thyroxine (fT4), free triiodothyronine (fT3), and high titers of thyrotropin receptor antibodies (TRAbs) (8). Excluded from the study were those with: systemic or other immune-medicated diseases; subclinical hyperthyroidism; previous GD relapse; Graves’ ophthalmopathy; toxic adenoma; toxic multinodular goiter; and cases coming from iodine deficient areas. Healthy children recruited from the general population and matched for age, gender, pubertal status, and socioeconomic status (SES) were also included as control subjects for statistical comparison. The inclusion criteria for the control group were: demonstration of normal serum TSH and fT4; negative antithyroid antibodies; and no past or family history of thyroid disease.

### Methodology

All participants underwent detailed medical histories and clinical examinations with special emphasis on age at onset of GD and its duration. Anthropometric measurements (height and weight) and vital signs were recorded. Body mass index (BMI) was calculated using the standard formula: BMI=weight (kg)/height (m)^2^. BMI was expressed as standard deviation (SD) scores (SDSs) to normalize for age and sex (9) using national growth reference data (10). Blood pressure was recorded and expressed as SDS to normalize for age and sex (11). Pubertal development was assessed by Tanner staging (12). Thyroid volume was estimated using ultrasonography (7.5-MHz linear array transducer) (GE Healthcare Bio-Systems, Milwaukee, WI, USA). Thyroid volume values were obtained by calculating the volumes of both lobes as follows: Lobe (mL)=Length x width x depth (mm) x 0.479. Thyroid volume was expressed as SDS on the basis of published references values for age and gender (13,14). Imaging data were reviewed by the same pediatric radiologist, who was blinded to the biological data.

### Laboratory Investigations

Blood samples were obtained at 8.00 a.m. after an overnight fast for estimation of serum concentrations of TSH, fT4, and fT3 (Immulite™ 2000 Third Generation, Diagnostic Products Corporation, Los Angeles, CA., USA). The reference ranges for thyroid hormones were as follows: TSH=0.4-4.0 mU/L, fT4=10.0-26.0 pmol/L, and fT3=3.5-5.5 pmol/L. The coefficients of variations (CV) for thyroid hormones were as follows: TSH=5.0 and 5.1% at concentrations of 4.0 and 10.0 mU/L, respectively; fT4=6.5% at concentrations of 10.0 pmol/L; and fT3=8.9% at concentrations of 3.5 pmol/L. Serum TRAb levels were measured with a 3^rd^ generation TBII assay (TRAb3^rd^) using the automated Cobas electrochemiluminescence analyzer (Elecsys, Roche Diagnostics GmbH, Penzberg, Germany). The cut-off value for positive concentration of TRAbs was 1.75 IU/L. The serum concentration of high sensitivity C-reactive protein (hsCRP) was measured using an hsCRP enzyme-linked immunoabsorbent assay (ELISA) kit (catalog no. E29-056; Immunospec Corp., Canoga Park, CA, USA). Measurement of serum sCD40L levels was performed using a specific ELISA (Biosource Int., CA, USA) according to the manufacturer’s instructions. The intra-assay and interassay coefficients of variation for sCD40L were 5.00% and 6.30%, respectively, with a sensitivity of 0.067 ng/mL, The reference range for sCD40L level was 0.16-10 ng/mL (15).

### Ethical Consideration

The protocol of the study was carried out in accordance with the Declaration of Helsinki ethical principles for medical research involving human subjects. The study was approved by the Ethical Committee of Assiut University (approval number: 10/2018) and informed consent and assent were obtained from all participants or their parents/guardians for younger children before inclusion in the study.

### Statistical Analysis

All statistical analyses were carried out using Statistical Package for the Social Sciences, version 18.0 (IBM Inc., Chicago, IL, USA). Quantitative variables were presented as means±SDs, and qualitative variables were presented as percentages. The Kolmogorov-Smirnov test was used for assessing normality of data distribution. Comparisons between parametric and non-parametric values were performed using a two-tailed Student’s t-test and Mann-Whitney U tests, respectively. Categorical variables were compared using the chi-square or Fisher’s exact tests. Correlations between sCD40L and clinical, and laboratory variables were performed using Pearson’s correlation coefficient test. Multivariate analysis was used to determine the factors that were significantly associated with elevated sCD40L concentrations. The odds ratios (ORs), 95% confidence intervals (95% CI) and significances were calculated. For all tests, values of p<0.05 were considered statistically significant.

## Results

The study included 48 children, 34 girls (70.1%) and 14 boys (29.2%), with a mean age of 14.4±3.6 years (range: 11-18 years) with a new diagnosis of GD. Compared to 48 age, sex and SES matched healthy children, patients had significantly lower mean BMI SDS (p=0.01) and higher mean heart rate (p=0.01). Patients also had significantly higher mean hsCRP and sCD40L concentrations (p=0.001 for both) (see Table 1). Patients’ sCD40L levels had significant positive correlation with age (r=0.319, p=0.01), thyroid volume SDS (r=0.564, p=0.001), hsCRP (r=0.323, p=0.01) and TRAbs concentrations (r=0.632, p=0.001) but not with fT3, fT4, or TSH concentrations (see Table 2). Multivariate analysis showed that sCD40L concentrations were significantly correlated with TRAbs (OR=3.1, 95% CI: 2.2-2.7, p=0.001) and thyroid volume SDS (OR=2.1, 95% CI: 1.2-2.7, p=0.001).

## Discussion

The current study has demonstrated that sCD40L levels were significantly higher in children with GD compared with controls (p=0.001). Moreover, sCD40L correlated positively with TRAbs concentration, which remained significant after regression analysis (OR=3.1, 95% CI: 2.2-2.7, p=0.001). Mysliwiec et al (7) reported that sCD40L levels were elevated in adult patients with GD compared to control subjects, although the difference did not reach statistical significance. Experimental studies have shown in vitro that increased sCD40L concentrations were associated with adhesion molecules and monocyte chemoattractant protein-1 release, impaired migration of endothelial cells and O2 generation in monocytes (16) which suggested that that sCD40L played an important role in the regulation of autoimmune and inflammatory responses, which in turn are likely to be involved in the pathogenesis of GD (7). Blockade of the CD40-CD40L pathway with BI 655064 in rheumatoid arthritis patients with insufficient response to methotrexate-IR resulted in marked improvement in clinical and biological parameters (17,18), suggesting that the CD40-CD40L pathway might prove to be a target for novel therapeutic strategies for autoimmune diseases.

CRP is an acute-phase protein associated with systemic inflammation. In this study, the circulating levels of hsCRP were significantly higher in children with GD than in the control children. Furthermore, the hsCRP levels were positively correlated with sCD40L levels (r=0.323, p=0.01). These findings are consistent with those of previous studies (19,20), that showed increased systemic inflammation in adult patients with GD.

Interestingly age was significantly associated with sCD40L concentration (r=0.319, p=0.01). This is in agreement with El-Asrar et al (21) who reported significant positive correlation between sCD40L levels and age in a cohort of children with type 1 diabetes mellitus. On the other hand, Cholette et al (22) reported that sCD40L levels are high at birth and remain significantly higher throughout childhood than sCD40L concentrations in adults. Future research may help to answer questions regarding the underlying reasons for developmental changes in sCD40L serum levels.

Thyroid volume SDS was significantly higher in children with GD compared with control children (p=0.01). Furthermore, a significant positive correlation between sCD40L levels and thyroid volume SDS was demonstrated that reminded significant after regression analysis (OR=2.1, 95% CI: 1.2-2.7, p=0.001) suggesting a direct causal relationship between sCD40L and thyroid volume. Previous studies indicated that increased levels of sCD40L may reflect a greater degree of T cell infiltration of the thyroid gland in patients with GD as the degree of surface CD40 expression was shown to closely correlate with intensity of lymphocyte infiltration, in addition to the direct thyroid growth-stimulating role of sCD40L that may result in diffuse goiter (7).

On examination of the relationship between other markers of thyroid function and sCD40L there was a no correlation between sCD40L and either fT3 or fT4 concentration. This is in agreement with Yamamoto et al (23), who reported the same finding in adult patients with GD. Despite the possible important role of sCD40L in the pathogenesis of GD (24), it appears that high serum concentrations of sCD40L are associated with the presence of goiter but not with elevated thyroid hormone levels. However, further studies are needed to clarify the role of sCD40L in relation to the thyrotoxic activity of GD.

### Study Limitations

The cross-sectional survey and the small number of subjects represent the major limitations of this study. As such, it is not possible to conclude whether higher sCD40L levels are directly involved in the pathogenesis of GD or just a consequence of the immune-mediated process.

## Conclusion

This preliminary study has evidence of higher concentrations of sCD40L in children with newly diagnosed GD. There was also a strong positive correlation of sCD40L with both TRAbs and thyroid volume, which may suggest a biologically active role for sCD40L in the pathogenesis of GD.

## Figures and Tables

**Table 1:**
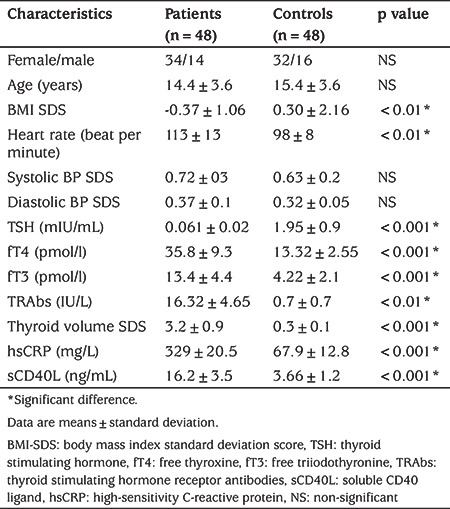
Clinical and laboratory characteristics of the patient and control groups

**Table 2:**
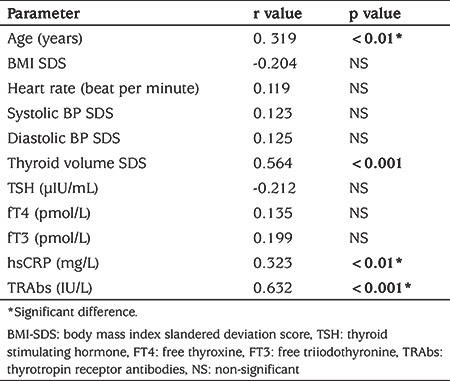
Correlation between soluble CD40 ligand and the other parameters in children with Graves’ disease
